# Prevention Efforts for Malaria

**DOI:** 10.1007/s40475-018-0133-y

**Published:** 2018-02-08

**Authors:** Tinashe A. Tizifa, Alinune N. Kabaghe, Robert S. McCann, Henk van den Berg, Michele Van Vugt, Kamija S. Phiri

**Affiliations:** 1 Training and Research Unit of Excellence (TRUE), Public Health Department, College of Medicine, University of Malawi, Blantyre, Malawi; 2 Academic Medical Center, University of Amsterdam, Amsterdam, Netherlands; 3 Wageningen University and Research Center, Wageningen, Netherlands

**Keywords:** Malaria, Sub-Saharan Africa, Vector control, Methods under development, Prevention in high-risk populations, Community mobilization

## Abstract

**Purpose of Review:**

Malaria remains a global burden contributing to morbidity and mortality especially in children under 5 years of age. Despite the progress achieved towards malaria burden reduction, achieving elimination in more countries remains a challenge. This article aims to review the prevention and control strategies for malaria, to assess their impact towards reducing the disease burden and to highlight the best practices observed.

**Recent Findings:**

Use of long-lasting insecticide-treated nets and indoor residual spraying has resulted a decline in the incidence and prevalence of malaria in Sub-Saharan Africa. Other strategies such as larval source management have been shown to reduce mosquito density but require further evaluation. New methods under development such as house improvement have demonstrated to minimize disease burden but require further evidence on efficacy. Development of the RTS,S/AS01 malaria vaccine that provides protection in under-five children has provided further progress in efforts of malaria control.

**Summary:**

There has been a tremendous reduction in malaria burden in the past decade; however, more work is required to fill the necessary gaps to eliminate malaria.

## Introduction

Malaria remains one of the most serious public health problems in lower and middle-income countries, and control and elimination are high priorities within endemic regions. Worldwide, an estimated 212 million new cases of malaria and 429,000 malaria deaths occurred in 2015 with 90 and 92%, respectively, occurring in the African region [1••]. Children under 5 years of age are particularly susceptible to infection in areas with high malaria transmission. More than 70% of all malaria deaths occur in this age group [1••]. Since the year 2000, a concerted campaign against malaria (Roll Back Malaria) has led to unprecedented levels of intervention coverage and scale-up of effective treatments across Sub-Saharan Africa (SSA) [2••]. Currently, the World Health Organization (WHO) has set new goals for global malaria reduction by 2030, which include the reduction of global malaria incidence and mortality rates by at least 90%, as well as elimination of the disease in at least 35 endemic countries [3].

As a communicable disease occurring mostly in the tropics and sub-tropic regions, malaria is caused by Plasmodium protozoan parasites. At least five parasite species are known to cause malaria in humans of which two are the most lethal: *Plasmodium falciparum* and *Plasmodium vivax* [1••]. The other parasite species include *P. malariae*, *P. ovale*, and *P. knowlesi*. *P. falciparum* malaria is a life-threatening infectious disease that is responsible for over 90% of malaria cases and almost all the malaria deaths worldwide [1••]. It is more prevalent in SSA and New Guinea. *P. vivax* is more prevalent is South America and Asia [4]. *P. malariae* is less prevalent and found in most endemic regions, particularly in SSA. *P. ovale* is also less common and is also found mainly in SSA [5]. *P. knowlesi* is the most recently discovered malaria parasite. It was discovered in its natural host, the long-tailed macaque, and is currently an important cause of zoonotic malaria in Borneo, Peninsular Malaysia, and beyond [4, 6].

Approximately 70 species of *Anopheles* mosquitoes are competent vectors of human malaria, with 41 of these species described as the “dominant” malaria vector species globally [7, 8] Three species within the *Anopheles gambiae* species complex, together with *Anopheles funestus*, are the principle vectors of malaria in most African countries [9, 10], while a higher diversity of vector species occurs in other parts of the world [7]. Anthropophilic, endophagic, and endophilic vectors, such as *An. funestus*, primarily take blood meals from humans indoors (and then rest indoors), making them efficient malaria vectors. Species that feed more opportunistically on both humans and livestock, such as *An. arabiensis*, or that bite or rest outdoors, are generally less efficient malaria vectors but can still be the primary vector species in a given region. These differences in ecology and behavior among vector species also lead to important differences in the effectiveness of vector control interventions.

The clinical manifestations of malaria vary with geography, epidemiology, immunity, and age. Understanding the complex pathogenesis of malaria requires exploring mechanisms of parasite invasion and host immune response. Symptoms of malaria include severe headache, nausea (vomiting), chills, and typical fever cycles. The severe manifestations often present clinically as cerebral malaria, pulmonary edema, acute kidney injury, hypoglycemia, lactic acidosis, anemia, and liver involvement [11]; severe malaria often occurs in individuals not previously exposed to malaria such as young children and travelers from non-endemic areas.

The control of vector-borne diseases such as malaria represents one of the global public health challenges of the twenty-first century. Successes of malaria prevention methods have been documented in several studies; however, there remains a gap in most settings in the use of these prevention methods. Much of the effort to control malaria has focused on the development of vector control strategies. Other protective mechanisms target high-risk groups such as pregnant women and young children. The main purpose of this review is to demonstrate efforts that have been made in the prevention and control of malaria, across the world, with a focus in under-five age group and in across all settings.

## Strategies Used in Malaria Prevention

### Vector Control

Vector control encompasses the measures that are directed against a vector of disease, intended to limit its ability to transmit the disease by protecting areas that are known to be receptive to transmission [12•]. Receptivity to malaria depends on the vectorial capacity of local vector populations, i.e., not just the presence of the vector but its population size, human biting habits, and longevity in relation to the period of sporogony [12•]. Each of these parameters is strongly influenced by the climate, local ecology, and behavior of both humans and vectors. Thus, vector control measures must match with the local setting to achieve optimum effectiveness. In an elimination phase, the objective of vector control is the reduction of the vectorial capacity of the local vector populations below the critical threshold needed to maintain transmission [12•].

### Main Vector Control Methods

1. Insecticide-treated mosquito nets (ITNs)

ITNs include long-lasting insecticidal nets (LLINs), where the insecticide lasts for up to 3 years, and nets conventionally treated, where the insecticide is active for up to 12 months. The WHO recommended that all health ministries and donor agencies scale up the distribution of ITNs, specifically to target populations of small children and pregnant women [13] since they are the population at risk. Most national malaria control programs have now adopted the universal coverage of ITN distribution, with mass distribution campaigns being conducted at intervals. Accordingly, out of the total 663 million malaria cases averted in the past 15 years in SSA, 67–73% has been attributed to the extensive distribution and use of ITNs [14]. The large expansion in the distribution of ITNs has been motivated by evidence from cluster-randomized controlled trials (RCT) that showed pooled relative reductions in child mortality of 18% [15, 16] and parasite prevalence of 20% from net use [15].

However, despite massive scale-up of ITN distribution in SSA, shortfalls and inequities exist [17], which compromise long-term elimination or control programs. A recent study in SSA, looking at equity trends in ownership of ITNs, showed that significant increase in ITN ownership has favored the poorest households in most settings; this has been attributed to the increase in national ITN distribution campaigns. Sierra Leone and Zimbabwe showed greatest improvement in equity [18]. There still exists a large gap both in terms of ownership and utilization. Ownership of ITN ranges between 34 and 98.4% of households at risk of malaria in SSA. A multi-country analysis of observational data in SSA looking at associations between ITN household ownership and child mortality as well as parasitemia prevalence showed that ownership of at least one ITN was associated with a pooled relative reduction of mortality in under-five children of 23% and a pooled relative reduction in parasitemia prevalence in children of 24% [19]. Another study in western Kenya showed that despite high mosquito net ownership, actual usage is still remarkably low, with different seasons showing remarkable variations [20]. In the study, an area called Emutete showed that the parasite prevalence in under-five children not using ITN and using ITN was 14 and 11%, respectively, during the rainy season. During the dry season, the parasite prevalence was 10% in under-five children not using ITN and 4% in those using ITN [20].

An estimated 47% of the population at risk of malaria did not sleep under a treated net in 2016 [11]. In general, possession of ITNs has been shown to be associated with factors such as proximity to distribution sites, cost, socioeconomic status, and the method of distribution. Among under-five children, possession is mostly affected by presence of health facility in the community, caregiver’s education, and residence as shown by a study in Northern Nigeria [21]. There is a low uptake in ITN usage in areas that are far from the distribution sites. In countries where ITNs are partially subsidized and socially marketed, cost is the main factor which prevents the poorest of the poor from accessing and using them [22, 23].

Several strategies need to be adapted to ensure adequate usage of ITNs. Conducting effective behavior change communication to change people’s mindsets towards bed net usage is one strategy that can be employed. Behavior change communication is used to encourage families to use their ITNs regularly, care, and repair them [24]. Apart from this, other related programs such as antenatal clinic attendance and intermittent preventive therapy in pregnant women (IPTp) uptake which ensure the well-being of pregnant women and infants are promoted using behavior change communication [24, 25]. Having proper maintenance strategies of ITNs and ensuring a steady supply in all endemic areas is another strategy; however, this is a major challenge but some desirable systems that have shown to work better in support of this strategy include targeting high-risk groups such as pregnant women and infants as shown in Tanzania. This is done by a school-based approach using vouchers targeting all students in primary and junior high school, combining it with the Tanzania National Voucher Scheme (TNVS) which distributes ITNs to pregnant women and infants. This appears to meet best the criteria of effectiveness, equity, and efficiency [26]. Repeated mass campaigns and some level of private sector delivery is another strong potential back-up strategy [26].

Socioeconomic status is a major influence of having knowledge and access to health care. Low socioeconomic status is associated with poor health-seeking behavior in general and specifically with low uptake and use of ITNs [22]. Although many health ministries and non-governmental organizations (NGOs) widely distribute ITNs for free or at low cost, their incorrect and inconsistent use remains problematic. Usage patterns differ among age groups [27], house construction, sleeping configuration [27, 28], and education level. In a review about a multi-country comparison of ITN use versus ITN access, using data from 41 DHS and MIS surveys (2005–2012) in SSA showed that over 80% of those with access to an ITN within their household reported using an ITN the previous night. These results differ from previous interpretations of the net use gap occurring as a failure of behavioral change communication interventions. The interpretations were not justified and that the gap was instead primarily driven by lack of intra-household access [29].

The second issue has to do with resistance to the pyrethroid insecticides used in ITNs. Pyrethroids are the only insecticide class approved for use on ITNs. Resistance to the pyrethroid insecticides in *Anopheles* malaria mosquito vectors is now widespread throughout SSA [30••]. There is growing concern that the expansion of pyrethroid resistance could jeopardize current momentum of reduction and elimination of malaria [31]. In a review looking at a data set containing data from 1955 to October 2016 from 71 malaria endemic countries, it has emerged that there is a rise globally in insecticide resistance. Insecticide resistance levels result in reduced efficacy in using ITNs as well as other vector control methods such as indoor residual spraying [32]. This extent of global insecticide resistance threatens the gains made in malaria control to date [31]. Therefore, there is need for the development of newer and effective insecticides to use as alternatives.

2. Indoor residual spraying (IRS)

Indoor residual spraying was the main strategy of the Global Malaria Eradication Campaign which resulted in the elimination of malaria from many countries and greatly reduced its burden in others [3]. In 2015, approximately 106 million people were protected by IRS [1••]. Generally, it has targeted areas with low and/or seasonal transmission, and its recent expansion into high transmission areas has been questioned due to concerns about long-term sustainability [3].

IRS has been used in several countries for malaria eradication and control of epidemics. Its effectiveness is well shown in several studies. For instance, in a RCT conducted in Tanzania looking at stable malaria cases (entomological inoculation rate (EIR) > 1), IRS reduced re-infection with malaria parasites detected by active surveillance in children following treatment. The protective efficacy (PE) on reducing re-infection with parasites was 54%. In the same setting, malaria case incidence assessed by passive surveillance was marginally reduced in children aged 1 to 5 years; the PE was 14%, but not in children older than 5 years in which the PE was − 2% [33••]. For unstable malaria (EIR < 1), in two RCTs, IRS reduced the incidence rate of all malaria infections; the PE was 31% in India and 88% in Pakistan.

Recently in Northern Uganda in a community-based trial carried around several districts, focusing on children under five, there was an increase in malaria cases after cessation of IRS. Incidence rate ratio in the under-five population rose from 0.77 in December 2014 (at the beginning of spraying) to 1.74 in June 2015 after spraying was ceased [34••]. Another study in Uganda during 4–18 months following discontinuation of IRS, absolute malaria test positivity rate (TPR) values increased by an average of 3.29% per month [35••].

IRS can be a lot costlier if used on a large scale in high transmission areas as it would require multiple spray rounds so that the population is protected. Another problem associated with IRS is that, as an intervention method, it has more risks to human health and the environment. Some insecticides such as dichlorodiphenyltrichloroethane (DDT), despite proving to be successful in controlling mosquitoes, are banned by some countries because of environmental hazards. It is recommended for use by WHO only under specific conditions. Similarly, like ITNs, insecticide resistance also poses a big threat in the use of IRS. Pyrethroid resistance is the main issue that is at hand.

3. Larval source management (LSM)

Larval source management (LSM) is the management of aquatic habitats that are the potential breeding sites for mosquitoes, to prevent the completion of immature development. It is one of the oldest tools in the fight against malaria but remains a largely forgotten and often dismissed intervention for malaria control in Africa [36]. With the recognition in recent years that outdoor biting contributes to malaria transmission [25], LSM has gained more attention, as it provides the dual benefits of reducing numbers of house-entering mosquitoes and those that bite outdoors.

LSM can be further classified into:

1. *Habitat modification*: Permanent change of land and water including landscaping, drainage of surface water, land reclamation, and filling. It is relatively easy and can be done in remote areas using simple materials [36, 37].
2. *Habitat manipulation*: Often recurrent, such as water level manipulation, includes measures such as flushing, drain clearance, shading, or exposing habitats to the sun. Like habitat modification, it is more suitable in resource-limited settings.
3. *Biological control*: Introduction of natural enemies into aquatic habitats (e.g., predatory fish, invertebrates, parasites, or disease organisms.) Predatory fish include *Gambusia affinis* [36, 37]. This method involves many resources and requires better organization from experts to facilitate this process.
4. *Larviciding*: Regular application of biological or chemical insecticides to water bodies to control mosquitoes. It is better in sites where habitats are few, fixed, and easy to find. Microbial larvicides have been proven efficient in the control of anopheline mosquito larvae and the reduction in adult mosquito densities. Safety advantage compared to chemical larvicide is that they are not harmful to other aquatic species. Access to microbial larvicides is still a challenge for developing countries due to cost, thus calling for development of alternative larval control strategies that can utilize locally available resources.

The most commonly used LSM method in many communities is larviciding [36]. A recent Cochrane review reported that larviciding in Sri Lanka of abandoned mines, streams, and rice paddies reduced malaria incidence by almost 75% compared to controls [37]. In five controlled before-and-after trials in Greece, India, the Philippines, and Tanzania, LSM resulted in an average reduction in parasite prevalence of around two thirds [37]. Current statistics from national malaria control programs show that only 34 malaria endemic countries worldwide use larval control in certain foci of malaria transmission [38].

### Methods Under Development

4. House improvement (HI)

Houses are not the only location where malaria transmission occurs, but they remain the most important transmission environment in many endemic areas [39••, 40, 41]. Historically, improved housing was thought to have contributed to the elimination of malaria in the USA and to its decline in Europe [42]. Modern houses tend to be more protective against malaria than traditional houses made of natural materials that leave multiple gaps through which mosquitoes can enter [43], and in some settings, they offer protection equivalent to ITNs [39••, 44]. Data from demographic and health surveys as well as malaria indicator surveys conducted in 21 countries in SSA between 2008 and 2015 has shown that modern housing reduces malaria prevalence compared to traditional housing [45••]. Across all surveys, modern housing was associated with a 9 to 14% reduction in the odds of malaria infection [45••]. The review showed that malaria prevalence measured by malaria rapid diagnostic test ranged from 0.3% (Senegal 2013–2014) to 61.2% (Burkina Faso 2010) among children living in modern houses and from 1.5% (The Gambia 2013) to 79.8% (Burkina Faso 2010) among children living in traditional houses [45••]. House improvement refers to the full screening or closing of openings such as windows, doors, and eaves or installation of ceilings. The goal is to reduce mosquito-human contact indoors. Thus, as one of the supplementary malaria control interventions, it helps to provide protection to malaria indoors. Even with outdoor biting and transmission, there is evidence that a mosquito is likely to enter a house at some point during its life prior to delivering an infective bite [46•]. This intervention can be greatly used in the rural areas as cheap and simple materials can be used in making the screens, closing eaves, and other openings. A Gambian study where 500 households in a village were assigned with full screening, screened ceilings, or no screening at all demonstrated that more mosquitoes were trapped in houses that had no screening compared to the rest of the houses. More cases of anemia about 19% (children aged 6–10 years) were also found in houses that had no screening at all. [44].

5. Sugar feeding

Attractive toxic sugar bait (ATSB) methods are a new form of vector control that kill female and male mosquitoes questing for essential sugar sources in the outdoor environment [47]. The ATSB approach uses fruit or flower scent as an attractant, sugar solution as a feeding stimulant, and oral toxin to kill the mosquitoes [47, 48]. The ATSB solutions are either sprayed on vegetation or suspended in simple bait stations, and the mosquitoes ingesting the toxic solutions are killed [47, 48]. This intervention is technologically and operationally simple, environmentally safe, and cheap, making it suitable for controlling malaria in lower to middle-income countries. ATSBs usually use spinosad and boric acid as insecticides, but recently, ivermectin has proven to be effective [48]. A study in the African-Syrian Rift Valley showed a 95% reduction in female *Anopheles sergentii* populations, while completely eradicating males. In return, malaria vectorial capacity reduced from a pre-treatment level of 11.2 to 0.0 [47]. In a separate study in Mali, ATSB treatment reduced densities of female and male *An. gambiae* sensu *lato*. by about 90%. After spraying ATSB in the treatment site, population densities of female and male *An. gambiae s.l.* declined rapidly over a week and then stabilized at low levels [48]. Applications of ATSB can reduce malaria vectorial capacity from relatively high to negligible levels which in turn results in reduced potential for malaria transmission to near zero levels [47]. This highlights the importance of ATSB as a promising new tool for outdoor vector control; however, further research is required in order to demonstrate its impact on disease burden and mortality.

6. Mass drug administration (MDA)

Mass drug administration is the treatment of the entire population in a geographic area with a curative dose of a drug without first testing for infection and regardless of the presence of symptoms [49]. It has been used for malaria control since the early 1930s and was advocated by WHO for malaria elimination and eradication in the 1950s [50]. MDA with antimalarials, in combination with other malaria control measures, has shown to be successful. For instance, MDA with sulphadoxine pyrimethamine combined with IRS achieved high levels of malaria control during the Garki Project in Northern Nigeria in 1969 [51]. A combination of primaquine with chloroquine was given to an estimated 70% of Nicaragua’s population preventing an estimated 9200 cases of malaria [52].

In recent developments, mass drug administration with ivermectin is proving to be a success in malaria control, especially for residual malaria. Ivermectin is an endectocide currently approved for human use. It is a semi-synthetic derivate from the fermentation products of *Streptomyces avermectinius*. It is one of the few drugs used in human mass drug administration (MDA) campaigns, and more than one billion treatments have been delivered over the last 25 years for controlling neglected tropical diseases such as onchocerciasis, strongyloidiasis, and lymphatic filariasis [53, 54•]. The drug when administered remains in the blood stream for approximately 6 days and causes blood to become toxic for malaria mosquitoes. This in return reduces the survival of Anopheles mosquitoes that feed on an ivermectin-treated person after a single standard oral dose [53, 54•]. Mosquito survival assessments done after annual ivermectin MDAs in Senegal, Liberia, and Burkina Faso demonstrated that ivermectin MDA reduces wild *An. gambiae* survivorship by more than 33% for up to 6 days, and the sporozoite rate was reduced by over 77% in the following 2 weeks following MDA [54•]. There is still a knowledge gap however on the expected clinical and public health impact on the use of ivermectin; hence, there is need for further research to show this. The major drawbacks of MDA are that it is mainly costly to persuade the patient population to take the drugs such that it requires repetitive and adapted health promotion activities. It also becomes difficult to do MDA in nomadic populations such that there should be need to adequately locate the whereabouts of these populations.

7. Swarm sprays

This is another vector control method that has been underexploited [55]. The locations of mating swarms are stable over the seasons [56] and appear linked to swarm markers on the ground, such as wells, wood piles, or the limits between footpaths and grass [57, 58]. A recent field trial conducted in Burkina Faso recruited a team of 20 volunteers from a village and targeted 300 swarm locations, spraying swarms with aerosols as they appeared over a 9-day period. These spray treatments reduced mosquito (*An. gambiae s.l*) density by 80% over a period of 10 days compared with a control village. It caused a significant reduction in the female insemination rate [59]. Like many other methods that are still under development, there is still more that needs to be done in order to measure its impact on disease burden.

8. Targeting livestock

Most malaria vector species exhibit a more diverse behavior, feeding on livestock as well as humans [39••]. Mosquitoes feeding on livestock could be targeted through treatment of livestock structures (e.g., IRS of cattle sheds) [39••]. Direct treatment of cattle with insecticides by dipping, sponging, or spraying has also been shown to kill mosquitoes [60, 61] and to reduce malaria in the human population [62]. Use of systemic veterinary insecticides that affect the mosquitoes upon blood feeding is another alternative for killing malaria mosquitoes. Ivermectin has been successfully tested in cattle and demonstrated to both kill mosquitoes and shorten the lifespan of survivors [54•, 63]. The major drawbacks are that not all livestock are treatable or have defined housing structures, and there is need for longer lasting endectocides to reduce treatment frequency and the issue of cost, as it can be expensive. There is also need for more studies to show the impact on minimizing disease burden.

9. Spatial repellents

Spatial repellents have been researched for many years and have demonstrated to have the potential of reducing transmission [64]. A feature of spatial repellents is that they can potentially provide protection in the evening before householders go to sleep and so could be complementary to ITNs [39••, 65]. One of the operational challenges, and the subject of ongoing research, is the development of long-lasting formulations or delivery systems to increase user acceptance and cost-effectiveness [39••, 64, 66]. However, the use of available consumer products (coils, vaporizers, impregnated mats, etc.) has been correlated with lower risk of malaria at the household level, depending on transmission environment and socioeconomic status [39••, 67].

10. Genetically modified mosquitoes

Genetic modification of Anopheles mosquitoes, by making them unable to reproduce or genetically resistant to malaria followed by driving these to the population, has for many years been a topic of discussion though it looked impossible. However, recent developments such as development of the CRISP/cas9 gene editing technique have the potential to transform this dream into reality. There are however many safety and ethical challenges to be met but this approach to malaria control is becoming a realistic option [68].

Despite the wide number of promising control tools tested against mosquitoes, current strategies for malaria vector control used in most African countries are not sufficient to achieve successful malaria control. Most national malaria control programs in Africa still rely on IRS and ITNs [69]. These methods reduce malaria incidence but generally have little impact on malaria prevalence [69]. Outdoor malaria transmission needs to be addressed by most African countries. The best way to approach malaria control is the use of integrated vector control management (IVM). This can be shown by a study done in Muleba district, Tanzania, where a combination of ITN and IRS resulted in an average prevalence of infection in children (0.5–14-year-old) of 13% compared to an arm that had ITNs alone where the average prevalence was 26% [70]. The combination of several vector control interventions helps to optimize the efficacy, as the vector can be controlled in its various stages of life and there can be an increase in the cost-effectiveness of vector control [12•, 71].

## Prevention of Malaria for High-risk Populations

Vulnerable groups in malaria endemic countries should be offered some level of protection against malaria. Some of the vulnerable groups include pregnant women, under-five children, nomadic populations, and visitors to endemic countries. Malaria in pregnancy remains a major public health problem in endemic areas of the African region where approximately 25–30 million pregnant women are at risk of *P. falciparum* infection and its adverse outcomes during pregnancy [72]. Most SSA countries use intermittent preventive therapy in pregnant women using sulphadoxine pyrimethamine (IPTp-SP) at each scheduled antenatal visit after the first trimester [38].

This chemoprevention has been shown to reduce maternal anemia, low birth weight, and perinatal mortality. In a recent systematic review and meta-analysis using trial registries and published studies on IPTp-SP from their inception to 2012, there were few cases of low birth weights in pregnant women who received at least three or more doses of SP during the gestation period compared to those that received less or none [73]. This group also had less placental malaria and a reduced risk of moderate to severe anemia [73]. In a few settings, due to the development of resistance of SP, lumefantrine artemether has been used for chemoprevention in pregnancy [38].

Intermittent preventive therapy in infancy (IPTi) with SP is a full course of antimalarial medicine that has shown to reduce clinical malaria, anemia, and mortality. A pooled analysis of data from six completed trials on IPTi with SP demonstrated 30% protective efficacy against clinical malaria and 21% PE against anemia in the first year of life [74]. Despite this, uptake of IPTi has been a challenge in many malaria endemic countries. This can be attributed to lack of awareness of IPTi in many malaria endemic countries and it is not properly integrated with other childhood programs such as Expanded Program on Immunization (EPI) [75]. By 2015, no countries had reported implementing this policy. At present, only Sierra Leone is implementing the policy on a pilot basis [38].

Seasonal malaria chemoprevention (SMC), which is normally administered by community health workers, is the presumptive use of SP in combination with amodiaquine (AQ) at monthly intervals to children aged 3 to 59 months in areas of acute seasonal transmission. This policy has been adopted mostly by countries in the Sahel sub-region. Stepped-wedge randomized cluster trials involving fifty-four health posts conducted in central Senegal from 2008 to 2010, in children aged 3–59 months, showed that there was a reduction of 60% in the incidence of malaria cases confirmed by a rapid diagnostic test (RDT) and a reduction of 69% in the number of treatments for malaria. Further, the reductions in the prevalence of parasitemia at the end of the transmission season in SMC areas were 68% in 2008, 84% in 2009, and 30% in 2010 [76].

Though still under development and some in trials, malaria vaccine shows progress in efforts of eliminating malaria. Currently, RTS,S/AS01 (known as “RTS,S”) is the most advanced form of the vaccine. In a number of randomized controlled trials in infants in SSA, RTS,S/AS01 vaccine provided protection against both clinical and severe malaria in African children [77]. The protective efficacy ranges from 20 to 30% depending on age. A pilot program of the vaccine in three countries in SSA (Malawi, Kenya, and Uganda) is currently underway, mobilized by the WHO [1••].

## Community Mobilization

Community mobilization and behavioral change mechanisms are significant for the success of all malaria prevention activities. This can occur in the form of public health communication, i.e., use of information, education and communication (IEC) materials, media, and community-based activities. Sometimes, using people with influence in the community and educating them on the benefits and correct use of malaria prevention tools helps communities to understand better about the disease. Efforts should focus on correcting misconceptions about malaria transmission, prevention, and getting prompt diagnosis treatment once one is suspecting signs and symptoms of the disease. Communities can also participate in vector control methods, for instance, in Ruhuha sector, Bugesera district, Eastern province of Rwanda, a community-based program demonstrated the feasibility and effectiveness of active community participation in malaria control activities since 2012. The baseline household survey in 2013 showed an increase from 94.5 to 98.7% in the coverage of IRS of the area [78]. A larval source control program was implemented in the area (intervention and control) with the community taking a lead role. Entomological monitoring and evaluation surveys showed a reduction in adult mosquito density in treated sites compared to the control [78]. Hard to reach areas are better placed to benefit from such activities as it is quite difficult to get resources to these areas.

## Conclusions

Malaria remains a public health concern particularly in SSA, causing so much mortality and morbidity especially among the pediatric population. Tremendous progress has been made over the last decade in reducing morbidity and mortality from malaria. The full impact of vector control has not yet been achieved and it needs urgent strengthening. ITNs and IRS are regarded as the key malaria prevention and control interventions because of their proven impact on reduction of disease burden; however, scaling up the combination of vector control strategies justifies in setting goals for malaria elimination in many countries. Development of new vector control methods is significant for malaria elimination; however, there are several gaps in most of these methods especially on the reduction of disease burden, requiring further research. Cost of this is the main issue making IVM a missed opportunity in so many endemic countries. Protection of high-risk groups such as pregnant women and children using antimalarials also reduces disease burden in endemic countries. There is need however to evaluate the general perception of populations towards the usage of IPTi as only one country is implementing it. The development of the malaria vaccine has proven to reduce the disease burden in under-five children; however, more evidence is required on its efficacy. The major setback in malaria prevention and control is mainly to do with drug and insecticide resistance. There are economic benefits attributable to malaria control activities as a lot of money can be saved and directed to other development activities. Efforts need to be put in place towards community mobilization in malaria control activities, monitoring programs and development of new control strategies to effectively control malaria. In general, combined efforts are required from all the stakeholders including governments, NGOs, research institutions, and scientists to come up with better strategies to combat malaria. This would result in ending preventable deaths of under-five children from the disease by 2030 as stipulated by the Sustainable Development Goals.
